# COVER: conformational oversampling as data augmentation for molecules

**DOI:** 10.1186/s13321-020-00420-z

**Published:** 2020-03-18

**Authors:** Jennifer Hemmerich, Ece Asilar, Gerhard F. Ecker

**Affiliations:** Department of Pharmaceutical Chemistry, University of Vienna, Althanstr 14, Vienna, Austria

**Keywords:** Deep learning, Toxicity, Imbalanced learning, Upsampling

## Abstract

Training neural networks with small and imbalanced datasets often leads to overfitting and disregard of the minority class. For predictive toxicology, however, models with a good balance between sensitivity and specificity are needed. In this paper we introduce conformational oversampling as a means to balance and oversample datasets for prediction of toxicity. Conformational oversampling enhances a dataset by generation of multiple conformations of a molecule. These conformations can be used to balance, as well as oversample a dataset, thereby increasing the dataset size without the need of artificial samples. We show that conformational oversampling facilitates training of neural networks and provides state-of-the-art results on the Tox21 dataset.

## Introduction

New regulations such as the EU regulation (EC) No 1907/2006 (widely known as REACH) make it complicate registering new chemicals on the market. Higher safety standards are important but also require more tests to be conducted. This is contradictory to the 3R principles of reducing animal testing experiments [1–3]. Additionally, over the last years pharmaceutical industries have faced a decline in newly marketed drugs [4, 5]. The concept “fail early, fail cheap” is gaining increasing importance since every failure in late stages of drug development is associated with high costs [6, 7]. The field of toxicity predictions was accelerated, when in 2007 the OECD published guidelines on the design of predictive models [8]. Since the 2016 release of a guideline allowing the mutagenicity assessment of drug impurities with in silico tools [9], in silico toxicology starts to turn from vision into reality. Reliable computational models could be of assistance in the early indication of hazards emanating from potential drug candidates [10]. Unlike in vitro testing, in silico approaches do not require the synthesis of a compound due to working with virtual molecules.

Currently, computational toxicology faces the problem of often having to deal with small imbalanced datasets (that is, with a high ratio between classes) which are hard to use for the training of models [11]. For toxicity predictions, especially if used in early stages of drug development, it is crucial not to miss potential hazards, but simultaneously not to predict hazards for every compound either. In the language of modeling, this means a model has to achieve high sensitivity and specificity at the same time. As these two are closely related, there will almost always be a trade-off. Nevertheless, for the best outcome, both properties should be maximized. This is especially challenging when using imbalanced datasets.

Due to the high ratio of classes in imbalanced datasets, the overall model has a high accuracy and area under the receiver operating curve (AUC). Yet, looking closer at the model often reveals a large gap between sensitivity and specificity. Mostly, classifiers are found to heavily lean towards predicting any new compound into the majority class. This problem arises firstly because the AUC is independent of the classifier threshold and thus does not reflect the current models’ performance. Secondly, the accuracy is strongly influenced by the majority class [12]. Subsequently, the performance of the model is overestimated. The immediate conclusion is to use appropriate metrics such as sensitivity and specificity itself [13] or the resulting balanced accuracy, which is calculated as the mean of the two. However, despite using the right metrics, sometimes models still fail to correctly predict the minority class. This is often due to the training error having been relatively small [14]. Furthermore, small datasets are prone to endorse overfitting, therefore needing a high amount of regularization on the network side.

Apart from the popular large datasets, the field of image recognition also faces the challenge of small datasets (e.g. [15–17]). For these cases the technique of data augmentation has proven to be very beneficial (e.g. [18–21]). In case of images, data augmentation refers to enriching the data set by applying different rotations, scaling, cropping or translations or filters such as Gaussian noise, all while preserving the labels. These techniques are well known to enlarge the training space as well as to reduce overfitting. Both properties are invaluable for neural network training with small datasets. In the field of cheminformatics Bjerrum, as well as Kimber and coworkers, showed that different SMILES can be used to augment the training data for a model predicting molecular properties [22, 23]. For regression models predicting the bioaccumulation factor, Sosnin and coworkers also used an augmentation with different conformations, however they do not report the comparison to models without augmentation [24].

In this paper, we propose a new method called COVER which facilitates model training on imbalanced chemical classification datasets. Our method uses Conformational OVERsampling (COVER) to generate distinct property vectors for the same molecule. This augmentation allows for balancing as well as oversampling of small and imbalanced datasets. Compared to training on SMILES, we hypothesize training a network on established molecular 3D properties requires a substantially reduced amount of abstraction by the network.

## Results

### Conformational oversampling

For validating COVER we used the Tox21 dataset [25]. The endpoint p53 activation (SR-p53) was selected as it is comprised of a high number of molecules and a fairly high imbalance ratio of 1:16. Overall, the Tox21 endpoints are well defined and, as seen in the challenge, lead to models with a high predictivity. After standardization and data curation, we calculated 3D-conformations of all molecules using RDKit. The base dataset (“1-1 dataset”) had one conformation per molecule. To verify our hypothesis that multiple conformations facilitate the training, we generated a series of additional datasets (see Table 2). First, we oversampled the minority class by 16, without also oversampling the majority class (“1-16 dataset”). Therefor, we calculated 16 conformations for the minority class and 1 conformation for the majority class, leading to a balanced dataset. Second, to evaluate whether enlarging the dataset adds additional value, we created larger balanced datasets. For this, we oversampled the majority class 2 or 5 times, followed by oversampling of the minority class 32 or 80 times (further referenced to as 2-32 and 5-80 dataset). To assess whether balancing is needed or whether increasing the dataset size would be sufficient, we generated two more datasets. For these, we oversampled both classes either 2 or 5 times, which are further referenced to as 2-2 or 5-5 dataset.

### Analysis of conformers

**Fig. 1:**
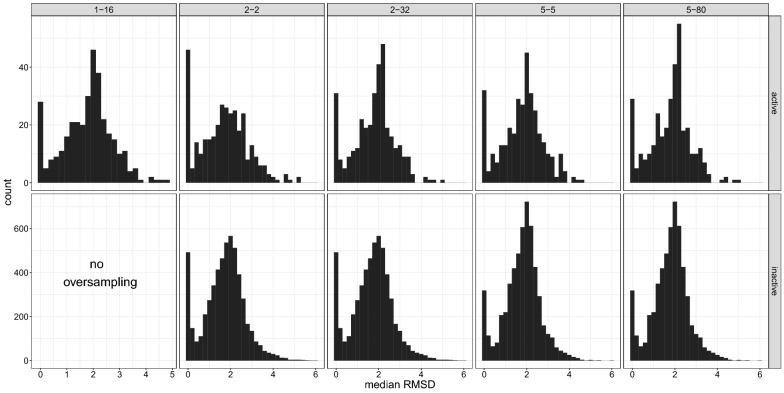
Histogram of the median RMSD of the generated conformations. The captions of the subplots denote the amount of oversampling done, e.g. 1–16 denotes 1 conformation for the negative class and 16 conformations for the positive class. The upper plots depict the distributions of the active molecules, the lower graphs indicate the distributions for the inactive molecules

**Fig. 2:**
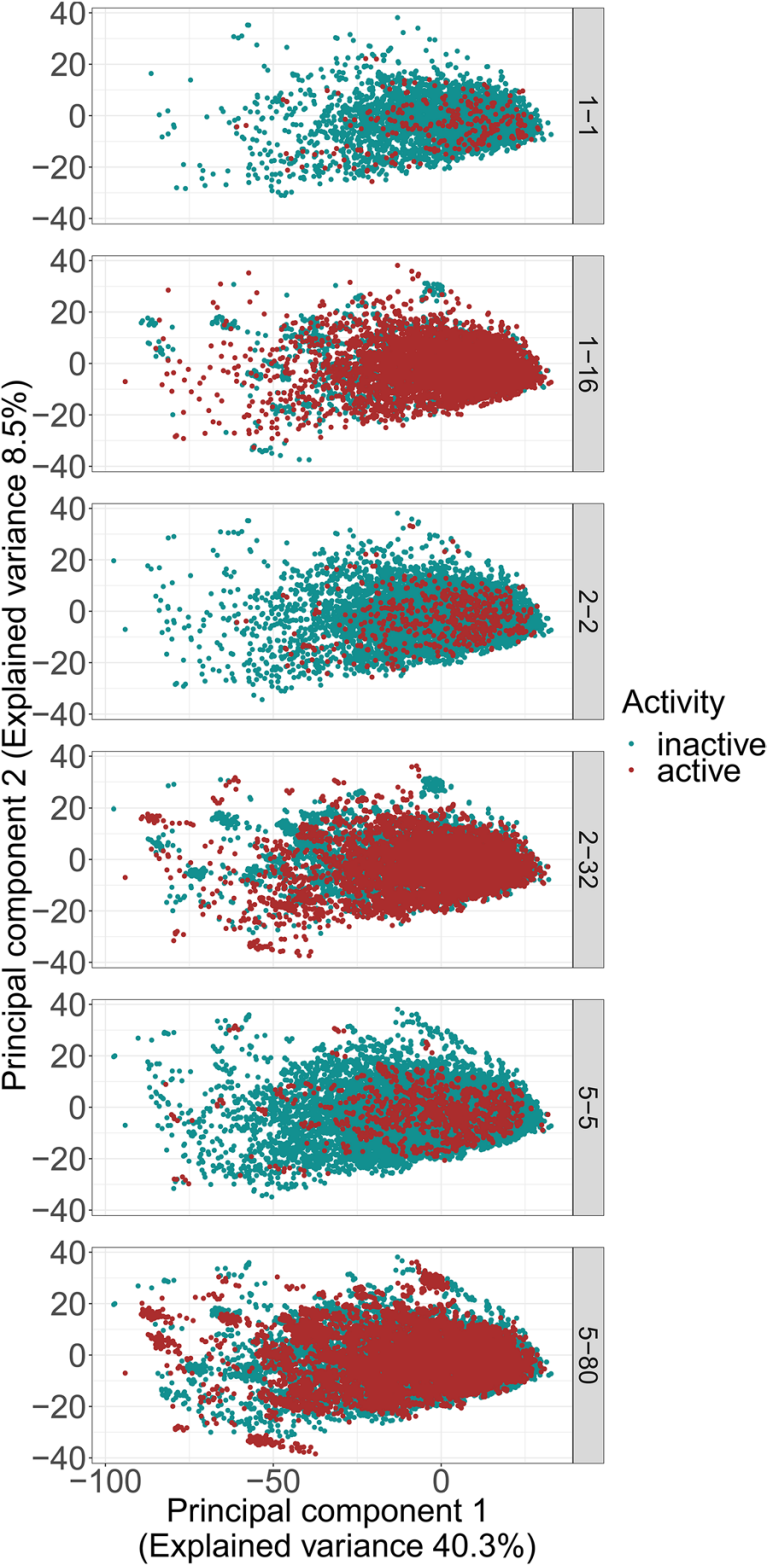
PCA of the oversampled datasets. Red dots indicate active molecules, green dots indicate inactive molecules. The captions of the subplots denote the amount of oversampling done, e.g. 1–16 denotes 1 conformation for the negative class and 16 conformations for the positive class

To generate conformations we used the ETKDG algorithm with UFF force field minimization developed by Riniker et al [26]. This algorithm ensures chemically reasonable conformations while maintaining diverse conformations. Nevertheless, the generation of a large number of conformations can yield duplicates or conformations with a small root-mean-squared deviation (RMSD). Therefore, we analyzed the RMSDs of the generated conformations. The histograms in Fig. 1 show the distributions of the median RMSDs per molecule follow approximately a standard distribution with a mean at around 2 Å. An exception is the pronounced peak of RMSDs between 0 and 0.2. This can be explained with rigid molecules present in the dataset, resulting in very similar or even identical conformations. Yet, in all cases the number of these conformations is below 10% of the overall dataset. The distribution of the median RMSDs also demonstrates that, although for rigid molecules considerably similar conformations are observed, generating conformations increases the diversity. The principal component analysis (Fig. 2) shows, the higher the number of conformations, the more space is covered by the dataset. Hence, oversampling appears to be beneficial by increasing the space which is spanned, especially by the active molecules.

### Model training

For each balancing or oversampling experiment we performed 3 independent runs (using different seeds for the network) for the nested cross-validation [27] scheme with hyperparameter grid search as described in the methods section. For each model, the grid search was run to prevent unsuitable model architectures which would impact model performance. To ensure that a good performance was not obtained by a “lucky” network initialization we conducted three runs with different seeds for each dataset.

**Fig. 3:**
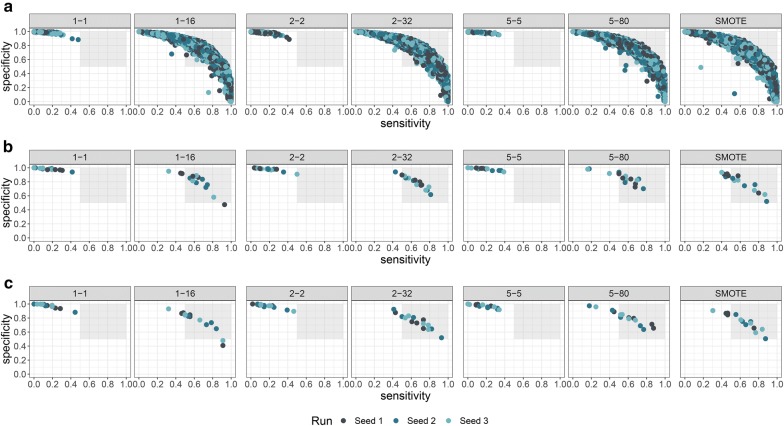
Sensitivity versus specificity of the trained models for differently seeded networks. Each dot presents the evaluation of one model. The captions of the subplots denote the amount of oversampling done, e.g. 1–16 denotes 1 conformation for the negative class and 16 conformations for the positive class. Each color denotes an independent run of the cross-validation scheme, using a different seed to initialize the network. The grey area indicates the area where sensitivity and specificity are greater than 0.5. **a** Plot of the models trained during the inner cross-validation and hyperparameter selection, **b** validation of the final models in the outer cross-validation loop, **c** validation of the final models on the external test dataset

Using the 1-1 dataset, we observed that training only on one conformation per compound yielded good results with respect to the AUC, but the balanced accuracy for 12 out of 15 models was lower than 0.6. Similar results were seen for the 2-2 and 5-5 dataset. In these cases, the models have a high specificity but lack sensitivity. For the balanced datasets, we observed a change in this pattern. Specifically, the models gained sensitivity with only a slight loss of specificity. The models for the 1-16, 2-32 and 5-80 datasets all achieved better performances in terms of balanced accuracy as compared to the other datasets. Figure 3a shows how COVER already impacts the hyperparameter search: The non-balanced datasets always achieved high specificity but lacked sensitivity, whereas the 1-16, 2-32 and 5-80 datasets show a wide range of model performances as would be expected during hyperparameter optimization. The wider range of model performances allows a hyperparameter selection based on the desired properties.

The outcomes for the external fold of the cross-validation can be seen in Fig. 3b. It shows the models trained by balancing the data do not suffer from low sensitivity. To ascertain that the models also work for an external dataset, we used the test set from the Tox21 challenge. None of the models showed a decrease in predictivity, with a similar pattern of increased sensitivity (Fig. 3c).

Interestingly, oversampling, in addition to balancing, only showed a slight increase in the balanced accuracy but no AUC increase. Generally the 95% confidence intervals, along with the means, show that increasing the number of conformations does not yield any significant change in the model performance but rather seems to introduce more variation (see Additional file 1: Figure S1 and Table S1). During training, we encountered fewer models in the upper left and lower right corner of the sensitivity-specificity plot, which are unfavorable regions due to very low sensitivity (lower right) or low specificity (upper left). To quantify the models further, out of 90 models which were built (3 $\times$ 5 models for 6 datasets) 58 had a balanced accuracy above 0.6, with 75% of them being trained on one of the balanced training sets. Overall, 37 out of the 58 models had the desired properties of a sensitivity and specificity above 0.5. All of these were trained with one of the balanced datasets. This further highlights training can be considerably improved by oversampling and balancing.

### Conformational independence

**Fig. 4:**
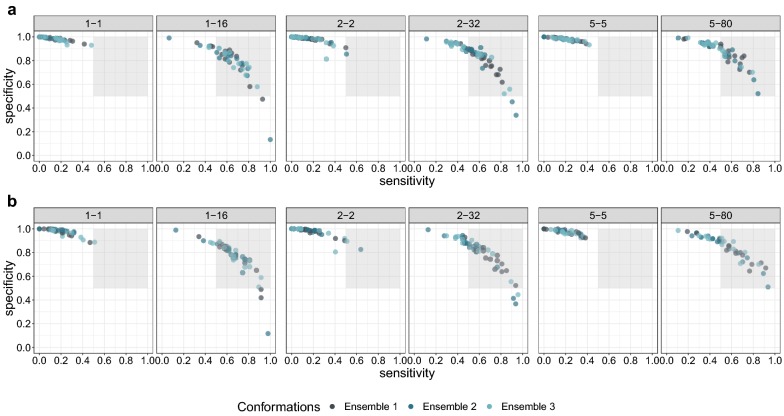
Sensitivity versus specificity of the trained models for different data ensembles. Each dot presents the evaluation of one model. The captions of the subplots denote the amount of oversampling done, e.g. 1–16 denotes 1 conformation for the negative class and 16 conformations for the positive class. Each color denotes an independent run of the cross-validation scheme, using a data ensemble with different conformations. The grey area indicates the area where sensitivity and specificity are greater than 0.5. **a** Plot of the models trained during the inner cross-validation and hyperparameter selection, **b** validation of the final models in the outer cross-validation loop, **c** validation of the final models on the external test dataset

In the first training step we evaluated the training for differently seeded networks to determine the independence from a specific “lucky” initialization of the network. Our results confirmed that conformational oversampling seems to be independent from the seed. However, it is also interesting to see the dependence on the provided conformational dataset. Therefore, we did an analysis on different datasets. To confirm the independence we used the final model architectures which we found performing the 5 × 4 cross-validation on one data ensemble (where data ensemble denotes one set of the 1-1, 2-2, 5-5, 1-16, 2-32 and 5-80 dataset generated with the same seed for the conformation generation) and retrained these models with two other data ensembles. Our hypothesis was, that, if the training is independent from the supplied conformations, the performance of the retrained models should be similar to the original models. Figure 4 shows that the oversampled models again outperform the models trained with unbalanced data. However, it can be observed that the performance of the original data ensemble is in general better as compared to the newly generated ensembles. This shows that the original model architectures are not yet fully optimized for the new datasets. Nevertheless, the oversampled datasets performed better in most of the cases, which underlines our hypothesis that the oversampling is independent of the supplied conformations. This is also indicated in the statistical analysis (see Additional file 1: Figure S1 and Table S1).

In addition to oversampling the training dataset, it is also interesting to evaluate the impact on oversampling on the test dataset. Since the model was trained on multiple conformations the model might be sensitive to the input conformation. To determine if the model performance increases by using an ensemble of multiple conformations, we evaluated our models with the Tox21 test dataset with 1, 5, 20 and 50 conformations per compound. However, we did not see any performance gain when using the mean prediction of multiple conformations (see Additional file 1: Figure S2).

### Comparison to synthetic minority over-sampling technique (SMOTE)

To further gain understanding and to compare COVER to existing methods, we chose the SMOTE algorithm [28] as it is very commonly used and very similar to COVER. The difference is that SMOTE creates synthetic examples by extrapolation to the nearest neighbours of a molecule, whereas COVER uses available information about the conformations of a molecule as an augmentation. For comparison we did a nested cross-validation using SMOTE to balance the 1-1 dataset from the data Ensemble 1 (see Fig. 3), and further used the hyperparameters found with Ensemble 1 for Ensemble 2 and 3 for an evaluation of the conformational dependence (see Fig. 4). In both figures the pattern observed for SMOTE is very similar to the patterns we observed for COVER. In the initial cross-validation as well as for the conformational independence a clear and very similar benefit of both, SMOTE and COVER can be seen as compared to training without oversampling. The descriptive statistical analysis also shows that COVER and SMOTE have a very similar benefit for model training (see Additional file 1: Figure S1 and Table S1).

### Comparison to the Tox21 challenge Top10

**Fig. 5:**
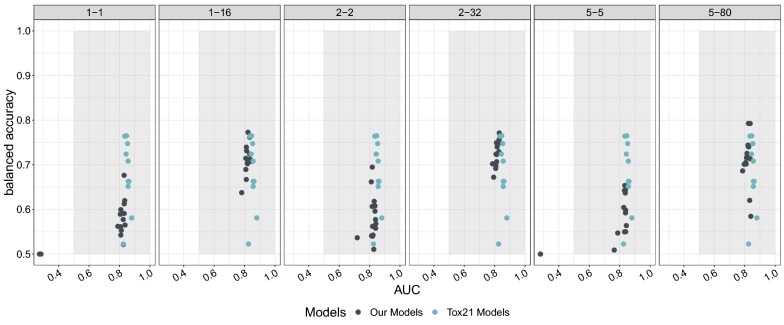
AUC versus balanced accuracy of the models for the differently seeded networks. Each dot presents the external evaluation of one final model. All models were trained with the same conformation ensemble but different seeds for the initialization. The captions of the subplots denote the amount of oversampling done, e.g. 1–16 denotes 1 conformation for the negative class and 16 conformations for the positive class. The grey dots represent the models trained by different oversampling strategies, whereas the blue dots represent the 10 best models as reported in the Tox21 challenge leaderboard. The grey area depicts the area where both, the AUC and balanced accuracy, are higher than 0.5

In the Tox21 challenge, neither sensitivity nor specificity were included in the analysis. Instead, balanced accuracy was used. The challenge leaderboard reports 10 models, ranked by their AUC (see also https://tripod.nih.gov/tox21/challenge/leaderboard.jsp). The winning model was an Extra trees classifier [29]. The models on ranks 2 to 6 were deep learning models [30]. The model ranked 7th had the highest balanced accuracy for this task, however, no detailed information can be found. Model 8 was an associative neural network [31], for 9 and 10 again, no information is available. For the challenge, the winning model had an AUC of 0.880 and a balanced accuracy of 0.581. The model with the best balanced accuracy has a balanced accuracy of 0.765 and an AUC of 0.847. Our model with the best balanced accuracy was a model trained on the 5-80 dataset. It achieved a slightly higher balanced accuracy of 0.784 with a slightly lower AUC of 0.803 (see also Table 1). The other models trained on the balanced datasets (1-16 and 2-32) had a slightly lower balanced accuracy of 0.760 and 0.753, with a similar AUC of 0.805 and 0.815 respectively (see also Table 1). For the non-balanced models the balanced accuracy was always about 10% lower than for the balanced models. A full comparison of our models with the top 10 models of the Tox21 challenge can be seen in Fig. 5. The performance of the different data ensembles also shows that the models can compete with state of the art models (see Additional file 1: Figure S3). However, the gain is not as visible as for the models trained with the data ensembles. Without a hyperparameter search, these models do not yet have a fully optimized architecture. Conclusively, these results also show that COVER is a viable method to oversample datasets leading to state-of-the-art results on the Tox21 endpoint p53 activation.

**Table 1:** Performance of the best models on the external test set

Dataset	Highest balanced accuracy		Highest AUC	
	Balanced accuracy	AUC	Balanced accuracy	AUC
1-1	0.664	0.811	0.610	0.814
1-16	0.760	0.805	0.678	0.818
2-2	0.680	0.801	0.561	0.821
2-32	0.753	0.815	0.753	0.815
5-5	0.630	0.814	0.551	0.823
5-80	*0.784*	*0.803*	0.693	0.823
SMOTE	0.740	0.813	0.740	0.813
Tox21 highest	0.765	0.847	*0.581*	*0.880*

The performance for the best single models trained with COVER and SMOTE as well as the best models for AUC and balanced accuracy from the Tox21 challenge.The best models are highlighted with italics numbers

## Discussion

Our approach shows that by balancing a dataset via creation of multiple conformations of a molecule, training of neural networks can be enhanced. Up to date, bootstrap aggregation [32] or SMOTE [28] are the techniques most widely used to balance chemical datasets. Bootstrap aggregation reduces dataset size, aggravating neural network proneness to overfitting. In this regard, SMOTE is much more suitable since it is also able to enlarge the training space of a dataset. However, this enlarged space is built up of synthetic instances. It was shown that these instances decrease the variance and can create noise by being created outside of the class decision boundaries [33]. Especially for highly imbalanced datasets, after oversampling with SMOTE, the minority class to a large extent consist of artificial samples. COVER is able to populate all areas of the available chemical space by retaining the inherent training set properties, without creating artificial molecular feature vectors. Although our method is shown to increase the training space, oversampling, in addition to balancing, does not substantially increase model performance. This might be due to the dataset’s inherent properties, since we do not add molecules to the dataset, but rather only broaden the recognition boundaries of molecules by presenting their variations. The sampling of conformations was derived from augmentations used in image based learning. In image recognition, the images are modified by cropping, adding filters, shearing and other transforming operations preserving the label. The network therefore is forced to learn a meaningful representation irrespective of the location (e.g. due to cropping) and exact appearance (e.g. due to added filters) of the object. Since the images are not linked, the network does not learn which image best represents the label, it merely is generalizing due to the modified, but increased, information. In our case, the network is supposed to learn meaningful combinations of descriptors to predict a molecules class. Just as the image network sees the modified images each as a separate training image, our network recognizes each conformation as a separate training molecule. Thus, we neither assume that the conformations are biologically relevant nor that the networks learn which conformation best represents the real world. They merely serve to increase the dataset and broaden the knowledge of the network, and in case of balancing, additionally reinforcing its ability to recognize the minority class. We could show that the performance of COVER is independent from the supplied conformations for the training, as well as, the test set. Whether a sophisticated conformation selection can further increase the performance should be investigated in the future.

Although we could not outperform the Tox21 leaders, our models achieved comparable results with one model achieving a slightly higher balanced accuracy. Regarding the time and effort reported, especially to build the winning deep learning models of the Tox21 challenge, our approach is less complex and requires substantially less time and tuning of different models. Additionally, combining the nested cross-validation scheme which we adapted from Baumann and Baumann [27] and the clustered cross-validation from the DeepTox pipeline [30] considerably helped to achieve state-of-the-art results. Using and external test set our models showed only a slight decrease in the performance. This is a strong indication of their good generalization. However, due to the clustered cross-validation we observed very different performances of the trained models, based on the current external validation fold. Observing a similar performance pattern for SMOTE confirmed that the fluctuations are results of the high dissimilarity between folds. It should be mentioned that the nested cross-validation approach is considerably more difficult with smaller datasets. Nevertheless, the Tox21 dataset proved to be an asset to thoroughly validate our approach by being able to cross-validate with the further possibility of an external test dataset.

In addition to the training procedure, it is worth pointing out the importance of the model reporting procedure. Whereas the AUC shows what a model is capable of, the actual model performance comprises only of a singular point on the AUC curve, depending on the chosen threshold. This is shown for our models on the 1-1 or 5-5 dataset, which can be seen in Table 1. Although we do see a very high AUC, the actual model with a standard threshold of 0.5 is incapable of detecting positives. Especially in predictive toxicology, this behavior can have detrimental effects. The goal for models generally is to be very sensitive towards potential hazards, but with a reasonable retainment of specificity. For a better performance of these models, the decision threshold would have to be determined outside of the model building.

## Conclusion

To conclude, with COVER we could show that the inherent information of chemical datasets is sufficient to generate models with state-of-the-art performance. By oversampling, using multiple conformations of the molecules, the models can utilize the full information of a dataset without reducing the dataset size or the creation of artificial samples. We envision that COVER will be a viable alternative to SMOTE and help to overcome the problem of imbalanced datasets in chemistry and aid in the training of better models.

## Materials and methods

### Used data

The original training data from the Tox21 challenge was taken from the DeepChem package and further processed [34, 35]. Data for the testing and evaluation set of the challenge was directly taken from the NIH homepage [25]. Data processing constituted of compound standardization, duplicate removal, and removal of compounds with ambiguous labels. Compound standardization included the following steps:

1. Split compound into disconnected fragments
2. Discard non organic fragments (not containing at least one carbon)
3. for each organic fragment do:
  Delete bonds to Group I or II metals
  Neutralize charges
  Apply rules for structure standardization (e.g. normalize functional groups, specific tautomers)
  Neutralize charges
  Remove compound if it is a solvent
  Generate InChI Key

The standardisation procedure was implemented in a KNIME workflow, using the RDKit [36] and the standardiser library (https://www.dev.ebi.ac.uk/chembl/extra/francis/standardiser/), for this work version 0.1.6 was used (https://github.com/PharminfoVienna/Chemical-Structure-Standardisation) [37]. The generated InChI Keys [38] are then used for searching and removing of duplicates. In case the labels of the duplicate compounds were matching, one copy of the molecule was kept. In case of mismatches, the compound was removed from the dataset. All steps of data curation were performed using a KNIME workflow (KNIME 3.6.1) [39] incorporating a python node for compound standardization (Python 2.7, Python standardiser library 0.19.0 and RDKit 2017.03.01). This procedure led to a final amount of 6112 compounds with 371 positives and 5741 negatives. For the test dataset, curation led to a total number of 733 compounds with 56 positives. 23 compounds present in the test and training dataset were removed from the test dataset.

### Clustered cross-validation

To train our models and ensure a low similarity between folds of the cross-validation, we used the clustered cross-validation approach as described by Mayr et al. [30]. For clustering, we used affinity propagation clustering [40] as implemented in the scikit-learn library (0.20.3) [41] with a pre-generated similarity matrix based on Morgan Fingerprints [42] folded to 1024 bits. The fingerprints were generated with a diameter of 4 using the RDKit library. Using this method, the number of clusters need not be chosen in advance. Due to the underlying algorithm, affinity propagation is capable of choosing appropriate exemplars and thus the number of clusters. After clustering, molecules belonging to the same cluster were distributed to the same, randomly chosen, fold. Consequently, molecules from the same cluster are always distributed/assigned to the same fold. Overall, this reduces the bias towards compound series in the dataset ensuring that the splits are as dissimilar as possible.

### Oversampling

**Table 2:** Datasets used for training with the number of molecules per class and the overall dataset size, each conformation is counted as separate molecule

Dataset	No. of conformations per		No. of molecules		
	Inactive	Active	Inactive	Active	Overall
1-1	1	1	5502	341	5843
1-16	1	16	5502	5428	10,930
2-2	2	2	11,001	680	11,681
2-32	2	32	11,001	10,865	21,866
5-5	5	5	27,504	1698	29,202
5-80	5	80	27,504	27,145	54,649

The oversampling was performed after the splits were generated. This ensures the oversampling only influences the training process, without introducing bias into the model. The oversampling was done using the conformation generation algorithm ETKDG from RDKit [26, 36]. We calculated the imbalance ratio as follows:

1 $$\begin{aligned} r = \Bigl \lfloor \frac{n_{maj}}{n_{min}} \Bigr \rceil \end{aligned}$$

With $n_{maj}$ being the number of majority class samples and $n_{min}$ being the number of minority class (i.e. positive) samples. After calculation r is rounded half up. This results in a final ratio of negatives to positives of 1:r. For the Tox21 data the imbalance ratio is hence 1:16. Subsequently, for the conformation generation, balancing the dataset we generated one conformation for each negative and r conformations per positive sample molecule. For additional oversampling, the ratio has to be multiplied by the desired number of samples for the negative class. So for each negative sample n conformations, and for each positive sample n*r conformations are generated. Overall, we generated six datasets. The first dataset has one conformation per molecule (1-1 dataset). The second dataset has 1 conformation for each negative and 16 conformation for each positive molecule, thus being balanced (1-16 dataset). Further, we generated two balanced datasets with 2 or 5 conformations per negative and 16 or 80 conformations per positive respectively (2-32 and 5-80 dataset), thus being datasets combining oversampling with balancing. Lastly, we generated two oversampled datasets without balancing. To achieve this we generated 2 and 5 conformations per molecule, irrespective of the class. The exact dataset sizes can be found in Table 2. After the conformations were generated we calculated 3D descriptors. In total, we used 117 3D descriptors available in the Molecular Operating Environment (MOE) software (Chemical computing group, https://www.chemcomp.com) and 1028 3D descriptors which use the internal compound coordinates available in the DRAGON 7 Software (Kode Cheminformatics, https://chm.kode-solutions.net/). Overall we generated each dataset three times with different seeds for the conformation generation algorithm. In the manuscript we will use the term data ensemble denoting a full conformation generation run, including a 1-1, 2-2, 5-5, 1-16, 2-32 and 5-80 dataset generated with the same seed.

### RMSD calculation and PCA

For investigation of the diversity of the generated conformations we used the Kabsch algorithm [43, 44] as implemented in the Chemistry development Kit (CDK) KNIME extension [39, 45]. Conformers were compared to all other conformers originating from the same molecule. This yields a triangular matrix with 0 on the diagonal and on the upper triangle. This matrix was used to calculate the median RMSDs per molecule. From these calculations we generated a Histogram to see the distribution, and hence the deviation of our conformations. Using the 3D descriptors we calculated a PCA with the prcomp function as implemented in R (version 3.4.4). Visualization for the Histogram of the RMSDs as well as the PCA plots was done using ggplot2 (version 3.1.1) [46].

### Training

To test our proposed approach, a 5 × 4-fold nested cross-validation scheme as proposed by Baumann and Baumann was used to train the models [27]. In this scheme two cross-validation loops are nested. The inner loop uses fourfolds to perform a fourfold cross-validation for hyperparameter tuning using a grid search. The best hyperparameter set was then used to retrain a model on all four inner folds. The remaining 5th fold was then used for model validation. With this procedure, in every run 5 models for different validation regions of the original dataset are produced, giving a better estimate for the model generalization. For each data ensemble (1-1, 2-2, 5-5, 1-16, 2-32 and 5-80 dataset generated with the same seed for the conformation generation) we did three runs with different seeds to confirm the independence from a specific seed. To validate further that the predictions are independent from a specific set of conformations, we did a thorough 5$\times$4 cross-validation for one data ensemble. In the following steps we assumed that, if the training is invariant to conformations, we can use the best model parameters for each dataset and retrain the model with the new dataset. Subsequently, the model performance should be similar to the performance of the data ensemble which was used to determine the network architecture. Thus for training with the dataset ensembles we used all final models from the previous training with different seeds. The networks were seeded similarly and trained with the respective hyperparameter set.

### Grid search

To train a model we used a grid search to find the best hyperparameters. Overall, each search trained 180 models with varying numbers of hidden units per layer, learning rate, dropout (input and hidden layers) and the number of layers. For the exact model parameters refer to Tables 3 and 4. To train the models we used early stopping [47]. The training was discontinued when we did not observe any increase in the balanced accuracy for 20 epochs.

**Table 3:** Parameter values used in the grid search

Parameter	Values
Learning rate	[0.01,0.1,1]
Hidden units	[256,512,1024,2048,4096]
Dropout input	[0,0.2]
Dropout hidden	[0.2,0.5]
Number of layers	[2,3,4]

**Table 4:** Fixed parameters for all networks

Parameter	Values
Activation	ReLu
Loss	Binary crossentropy
Optimizer	Stochastic gradient descend
Momentum	0.7
Initializer	He normal [48]

### Synthetic minority over-sampling technique (SMOTE)

For the comparison to SMOTE we used the same training and grid search protocols as for COVER. For training we used the initial 1-1 dataset with one conformation per molecule without any oversampling or balancing. For the grid search we used the same dataset we used for the grid search with COVER. To test the sensitivity to the initial conformations we used the same two datasets which were used to show the conformational independence of COVER. The oversampling was conducted during the cross-validation. Hence, after splitting the data into training and test set the training data was augmented. The augmentation was done without using the test dataset, as this would introduce bias. For SMOTE we used the implementation from the python package imbalanced-learn (version 0.6.1) [49].

### Model performance

Model performance is estimated using the comparison of the predicted values against the known activity. Tox21 labels are binary, 0 for negatives and 1 for positives. Since the last layer of a neural network is a sigmoid, predictions are given in the range of 0 to 1. A constant decision threshold of 0.5 was used for all experiments. This denotes that all molecules predicted above 0.5 were labeled as positives (1) and at 0.5 or lower a compound was labeled as negative (0). To calculate all metrics a confusion matrix was used. In consideration of the imbalance of our data and the goal of a predictive model for toxicity, we used sensitivity and specificity to evaluate our models. For training purposes, we optimized our models toward a high balanced accuracy, which is calculated as the harmonic mean between sensitivity and specificity. AUC was only used for comparison to the Tox21 data.

Sensitivity and specificity are calculated using the elements of the confusion matrix as follows:

2 $$\begin{aligned} sensitivity= \frac{TP}{TP+FN} \end{aligned}$$

3 $$\begin{aligned} specificity= \frac{TN}{TN+FP} \end{aligned}$$

With TP: number of true positives, FN number of false negatives, TN number of true negatives and FP number of false positives.

The area under the receiver operating curve (AUC) was calculated by plotting the true positive rate versus the false positive rate for varying decision thresholds and then calculating the area under this curve. The AUC was estimated by using the trapezoidal rule, implemented in the scikit-learn library (0.20.3) [41].

For the evaluation on multiple conformations we generated the mean of the predictions per compound and applied the threshold of 0.5 for a final classification of the compound. Subsequently the metrics were calculated as mentioned above.

### Implementation

Models were trained on two NVIDIA 1080Ti Graphics cards on a machine with 64GB RAM. The training was performed using the tensorflow [50] and keras [51] libraries. Parallel GPU training was conducted using the multi-GPU implementation from keras. Depending on the dataset size one full double cross-validation took between 72 and 120 h. Plots were generated using ggplot2 (version 3.1.1) [46].

### Descriptive statistics

To report differences between COVER, SMOTE and training without augmentation we calculated the mean, median, standard deviation, standard error and 95%-confidence interval using RStudio (version 1.2.5033) with R (3.4.4). As the output of a nested cross-validation is one model per fold, instead of first averaging over the models per run, we treated each model independently and calculated the standard deviation for all 15 models.

## Supplementary information

**Additional file 1.** Statistical analysis.

## Data Availability

The dataset can be obtained from the Tox21 challenge website as well as from the DeepChem library. The final curated training and test datasets with generated conformations, as well as the code used to train the models, is available at https://github.com/PharminfoVienna/COVER-Conformational-Oversampling.
