# Supplementary figures and images for: COVER: conformational oversampling as data augmentation for molecules
Source: J Cheminform. 2020 Mar 18;12:18. doi: 10.1186/s13321-020-00420-z (PMC7080709; doi:10.1186/s13321-020-00420-z)

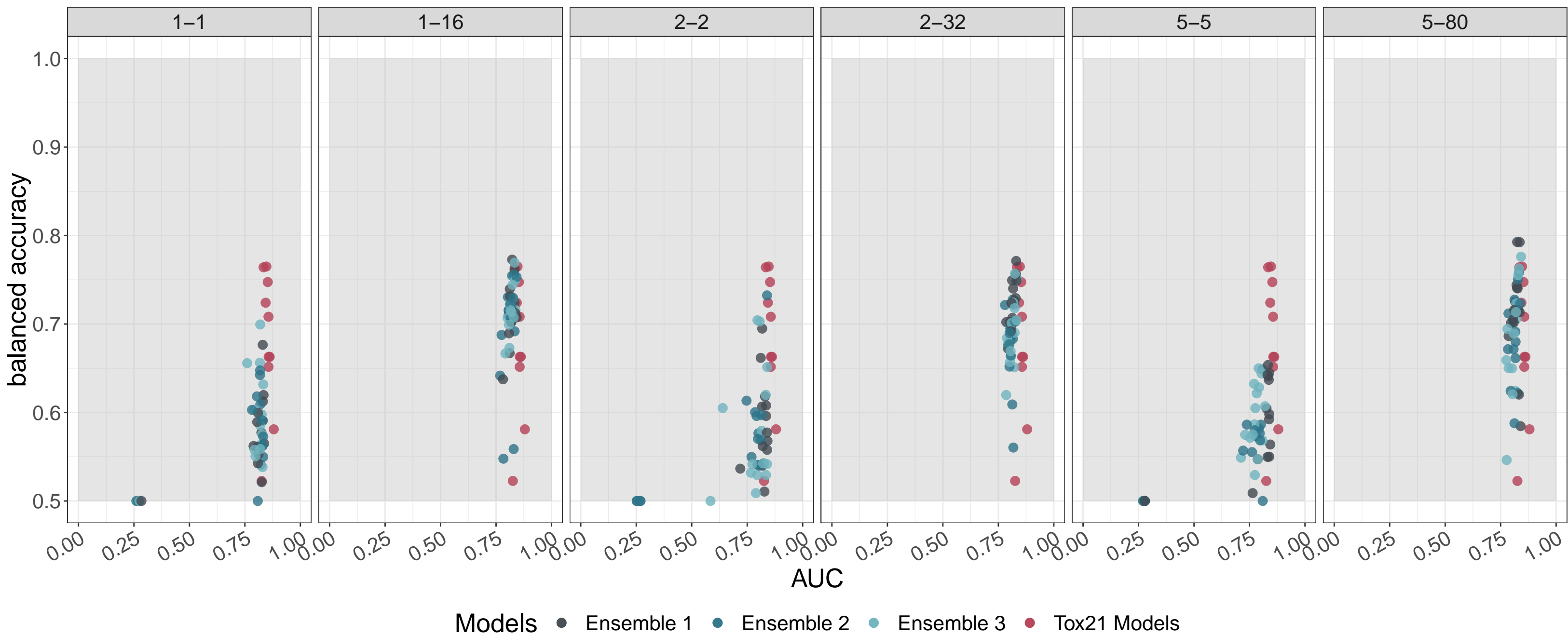

Supplement: Supplementary file 1 — Additional file 1. Statistical analysis. [file 13321_2020_420_MOESM1_ESM.zip › ComparisonTox21natconfs.pdf]

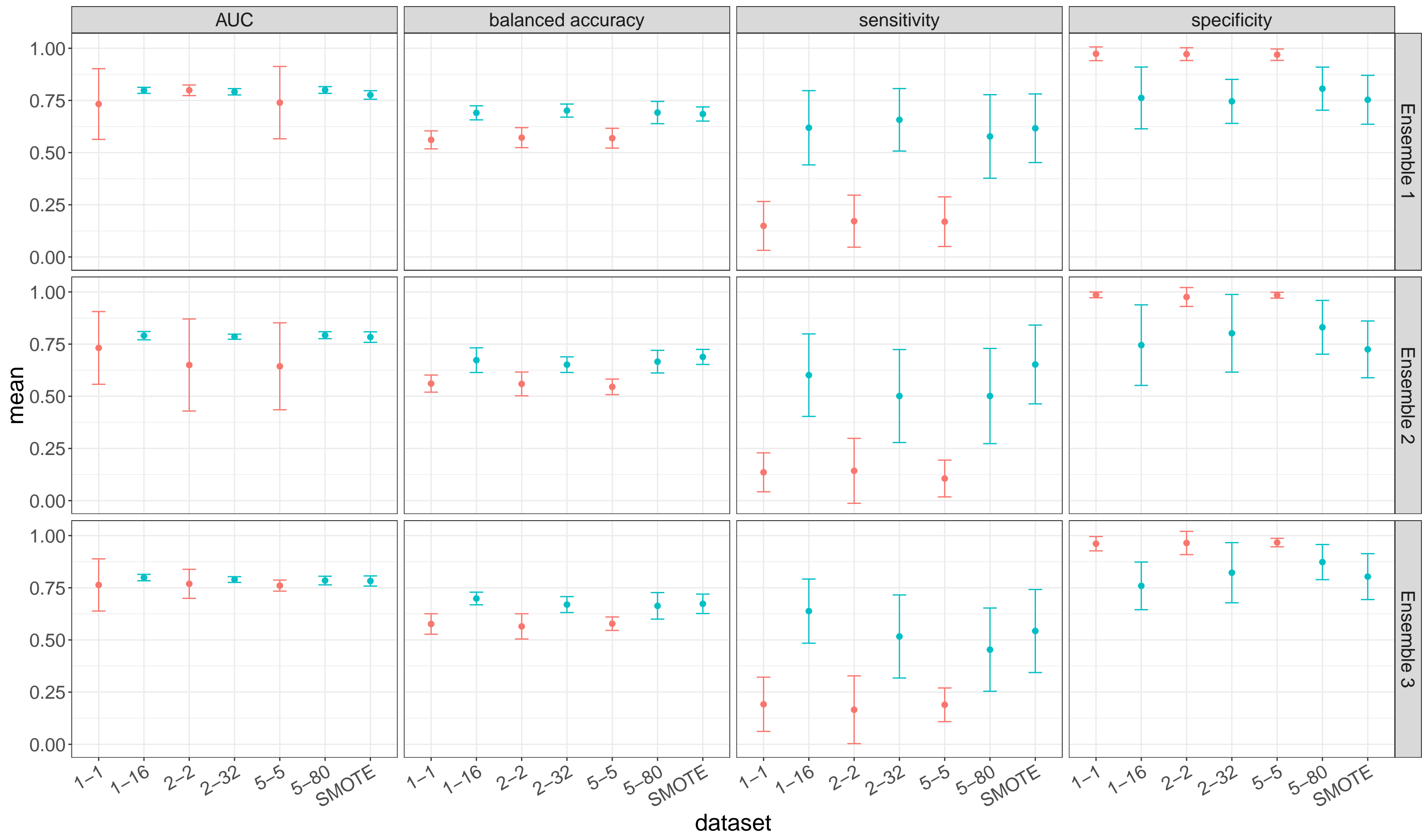

Supplement: Supplementary file 1 — Additional file 1. Statistical analysis. [file 13321_2020_420_MOESM1_ESM.zip › meansdalldatasets.pdf]

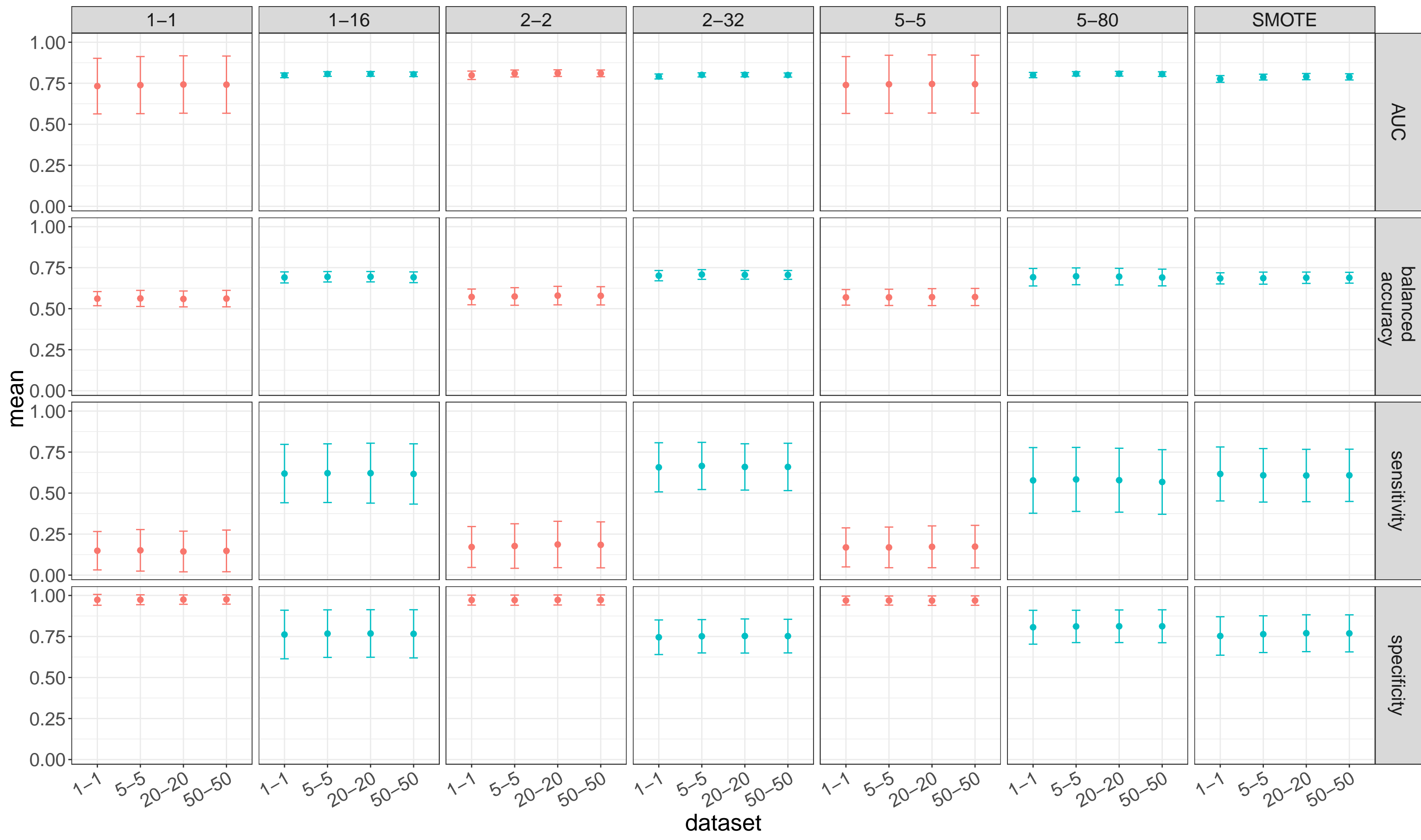

Supplement: Supplementary file 1 — Additional file 1. Statistical analysis. [file 13321_2020_420_MOESM1_ESM.zip › meansdalloversampled.pdf]
